# Whole Slide Imaging for High-Throughput Sensing Antibiotic Resistance at Single-Bacterium Level and Its Application to Rapid Antibiotic Susceptibility Testing

**DOI:** 10.3390/molecules24132441

**Published:** 2019-07-03

**Authors:** Donghui Song, Haomin Liu, Huayi Ji, Yu Lei

**Affiliations:** 1 Department of Biomedical Engineering, University of Connecticut, Storrs, CT 06269, USA; 2 Department of Chemical and Biomolecular Engineering, University of Connecticut, Storrs, CT 06269, USA; 3 Department of Computer Science and Engineering, University of Connecticut, Storrs, CT 06269, USA

**Keywords:** antibiotic resistance, whole slide imaging (WSI), antibiotic susceptibility testing (AST), minimum inhibitory concentration (MIC), single-cell growth rate analysis

## Abstract

Since conventional culture-based antibiotic susceptibility testing (AST) methods are too time-consuming (typically 24–72 h), rapid AST is urgently needed for preventing the increasing emergence and spread of antibiotic resistant infections. Although several phenotypic antibiotic resistance sensing modalities are able to reduce the AST time to a few hours or less, concerning the biological heterogeneity, their accuracy or limit of detection are limited by low throughput. Here, we present a rapid AST method based on whole slide imaging (WSI)-enabled high-throughput sensing antibiotic resistance at single-bacterium level. The time for determining the minimum inhibitory concentration (MIC) was theoretically shortest, which ensures that the growth of each individual cell present in a large population is inhibited. As a demonstration, our technique was able to sense the growth of at least several thousand bacteria at single-cell level. Reliable MIC of *Enterobacter cloacae* against gentamicin was obtained within 1 h, while the gold standard broth dilution method required at least 16 h for the same result. In addition, the application of our method prevails over other imaging-based AST approaches in allowing rapid and accurate determination of antibiotic susceptibility for phenotypically heterogeneous samples, in which the number of antibiotic resistant cells was negligible compared to that of the susceptible cells. Hence, our method shows great promise for both rapid AST determination and point-of-care testing of complex clinical bacteria isolates.

## 1. Introduction

Due to the overuse and misuse of antibiotics, the increasing emergence and spread of antibiotic-resistant bacteria in human infections, such as methicillin-resistant *S. aureus* (MRSA), vancomycin-resistant enterococci (VRE), extended-spectrum beta-lactamase (ESBL)- and carbapenemase-producers, has become a global threat to public health [1,2,3,4,5,6]. Unfortunately, the lack of newly developed antimicrobial agents has been worsening the crisis [7,8]. To reduce the inappropriate use of antibiotics, antibiotic susceptibility testing (AST) is employed by healthcare providers to guide the prescription of antibiotics. The most widely accepted AST methods, such as broth/agar dilution and Kirby-Bauer disk diffusion, are based on the observation of visible bacterial growth in the presence of antibiotics [9]. These conventional methods are routinely used and cost-effective, but they typically require at least 24–72 h for reliable readout [10]. Such delay leads to the empirical use of antibiotics and consequent increase in mortality [11,12]. Especially for patients with septic shock, initiation of inappropriate antimicrobial treatment results in a five-fold decrease in survival [13]. An alternative AST approach relies on the detection of specific genes or proteins responsible for antibiotic resistance by molecular techniques, such as polymerase chain reaction (PCR) and mass spectrometry [14,15,16,17,18,19,20]. Although molecular methods are sensitive and fast, the existence of biomarkers do not always correlate to phenotypic antibiotic resistance [21]. Furthermore, if new resistance mechanisms arise, they are likely to result in false negatives [22]. Thus, rapid AST techniques without compromising accuracy are urgently needed.

To date, a plethora of innovative approaches have been developed to shorten the time for AST to a few hours or less [23,24]. Particularly, several platforms employed microfluidics, optical imaging techniques or mass sensor arrays to determine antibiotic susceptibility by monitoring single-cell growth [25,26,27,28,29,30]. Considering that antibiotic resistant and susceptible cells coexist in clinical samples [31,32], apparently, detecting the rapidly multiplying individuals in a background of dying cells would take much less time than assays monitoring population growth. Thus, single-cell AST techniques would be more applicable for point-of-care tests. Nevertheless, since bacterial cells vary largely in their growth rates, the ability of available single-cell AST methods in providing precise results with statistical significance is hindered by low throughput. In a recent study, the throughput was significantly improved by analyzing the growth rates of thousands of single cells confined in microfluidic channels [28]. Although the concept introduced in this work is elegant, as clinical samples are typically polymicrobial and even genetically identical cells vary in size and shape [33,34,35], fixed dimensions of the channels would not be able to trap every single cell with various size and shape, thus losing potentially useful data concerning biological heterogeneity. In addition, since cells were confined to proliferate in only one direction and the ability of each cell to overcome the external restriction may vary, detection of growth rate using this method might not be as accurate as the results obtained from free-growing cells.

Here, we report a novel single-cell AST technique based on whole slide imaging (WSI) technique. Capable of digitizing a specimen on a glass slide into a single image, WSI has been extensively exploited to image histopathology slides for diagnostic use [36,37,38]. However, to our knowledge, the applications of WSI in AST have not been reported yet. In this study, WSI was employed to expand the imaging area, thereby allowing the visualization of a large population of cells regardless of their biological heterogeneity. As a demonstration, we conducted time-lapse imaging of thousands of *E. cloacae* cells in a WSI-compatible bacterial culturing glass slides that we previously reported [39] to track single-cell growth. Antibiotic susceptibility profiles of *E. cloacae* against gentamicin was determined by high-throughput analyzing single-cell growth rate. Moreover, to address the advantage of the significant improvement in throughput, our technique was applied to determine antibiotic susceptibility of an artificial phenotypically heterogeneous sample, in which the number of antibiotic resistant cells was negligible compared to that of the susceptible cells. For validation purposes, results obtained from our method were compared with the gold standard broth dilution method.

## 2. Results and Discussion

### 2.1. WSI-Based Monitoring Bacterial Growth at Single-Cell Level

To obtain an image encompassing a large population of single cells, “sandwich” slides with great bacterial cultivability that we previously customized for WSI was employed [39]. This system consists of a cover glass on the top, a supporting glass at the bottom and a 0.38 mm-thick gel pad with good optical transparency in between. Bacterial cells are immobilized on the interface between the gel and the supporting glass. Notably, owing to the “sandwich” structure and the refined composition of the gel, the gel pad is robust enough and tightly adhered to the supporting glass to avoid any deformation or sliding during WSI. Next, the phase contrast images of the entire sample area were acquired as the sample stage moves and seamlessly stitched together into one image.

**Figure 1:**
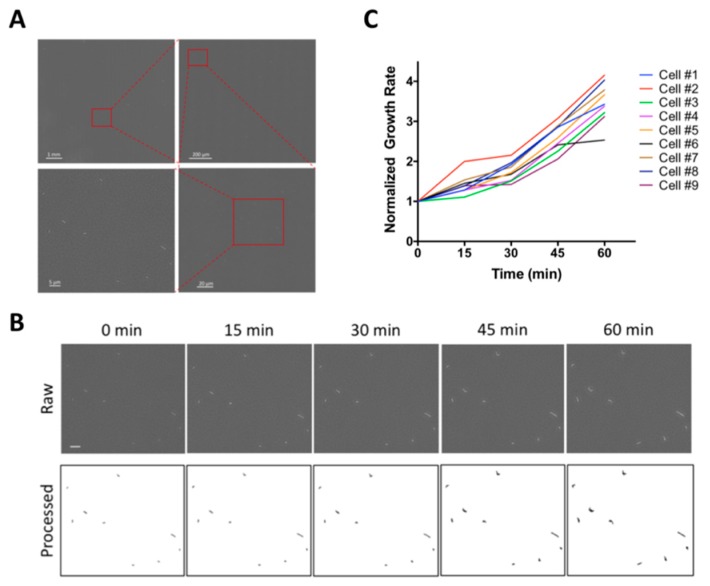
(**A**) A representative composite image captured by the reported platform. Zooming in the image allows the visualization of single bacterial cells. (**B**) Time-lapse images of one representative area selected from the composite image and corresponding post-processing binary images. Scale bar, 5 µm. (**C**) Single-cell growth rate of bacterial cells presented in (**B**).

To test the performance of this system, we conducted WSI to image *E. cloacae* cells deposited into the “sandwich” slides. Considering that the bacteria cells are small and might not sit on the same focal plane, rapid autofocus technology was applied to minimize out-of-focus issue. As a demonstration, Figure 1A displays a composite image of a sample area (8.48 mm × 7.69 mm). Zooming in the image enabled the visualization of individual cells, which were all in-focus. Herein, using a 40× objective lens, the imaging took only 3.5 min. Considering that the natural doubling time of bacteria is ~20 min and even longer during antibiotic treatment, it was assumed that there was no significant change in cell size during such fast imaging.

To employ this system to monitor single-cell growth, the “sandwich” slides were incubated at 37 °C and a humidity of 90%, under which condition the dimensions of the gel pad were not changed. Subsequently, time-lapse imaging was conducted on the same sample area at 15, 30, 45 and 60 min. After the composite images were collected, they were processed by ImageJ software and were converted into binary format images, in which the cells were black and the background was white. Since each composite image was in gigabytes, we selected a small subarea containing nine cells as a demonstration of analyzing single-cell growth (Figure 1B). To quantify the bacterial size, the area occupied by a single cell or the corresponding microcolony developed from it was measured from the processed images in Figure 1. Since there was no significant drifting of the x-y coordinates while the bacterial cells were growing, the areas with matched coordinates represented a group of bacteria originated from the same cell. For each individual cell, the normalized growth rate at time *t* was calculated using Equation (1), where *A_t_* and *A_0_* are the areas originated from the same cells at time *t* and time 0, respectively. Depicted in Figure 1C are the growth rate curves of nine cells. Apparently, at each time point, the variation of the growth rates was large, emphasizing the necessities of analyzing a large population of cells for the accurate determination of antibiotic susceptibility at single-cell level.

(1) $$Normalized\;growth\;rate=\frac{A_{t}}{A_{0}}$$

### 2.2. Determination of Minimal Inhibitory Concentration (MIC) by WSI-Based AST

**Scheme 1:**
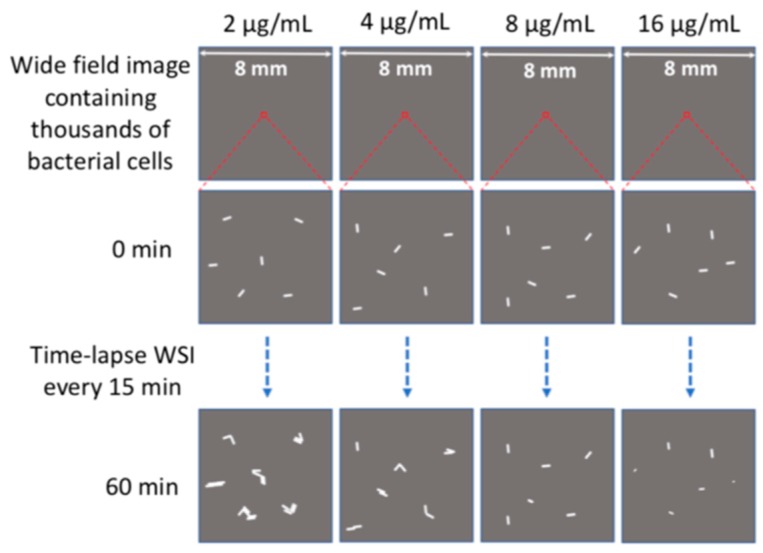
Schematic illustration of whole slide imaging (WSI)-based approach for rapid antibiotic susceptibility testing (AST).

**Figure 2:**
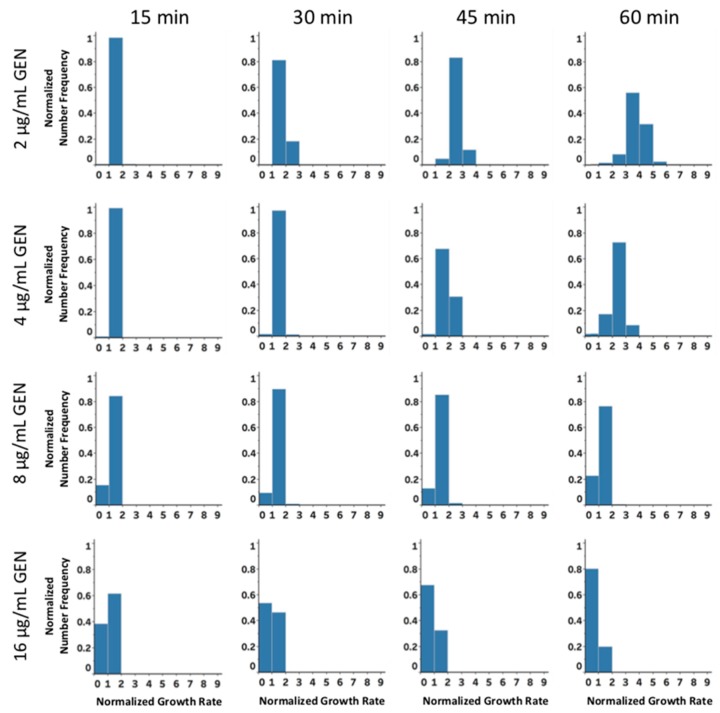
Number-based distribution of normalized single-cell growth rate for several thousand *E. cloacae* cells treated with 2, 4, 8 and 16 µg/mL of gentamicin.

**Figure 3:**
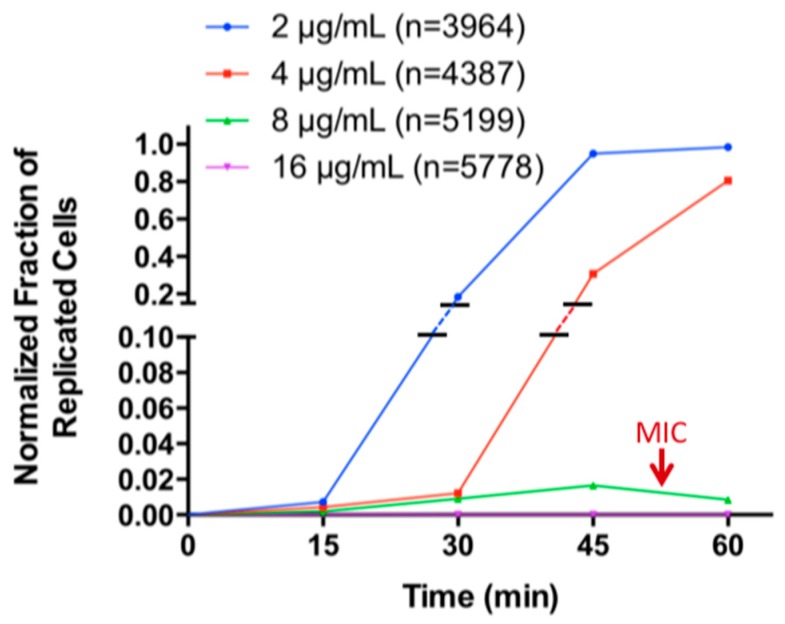
Minimal inhibitory concentration (MIC) determination by the reported approach. The curves were plotted by the normalized fraction of replicated cells from each treated group versus time.

**Figure 4:**
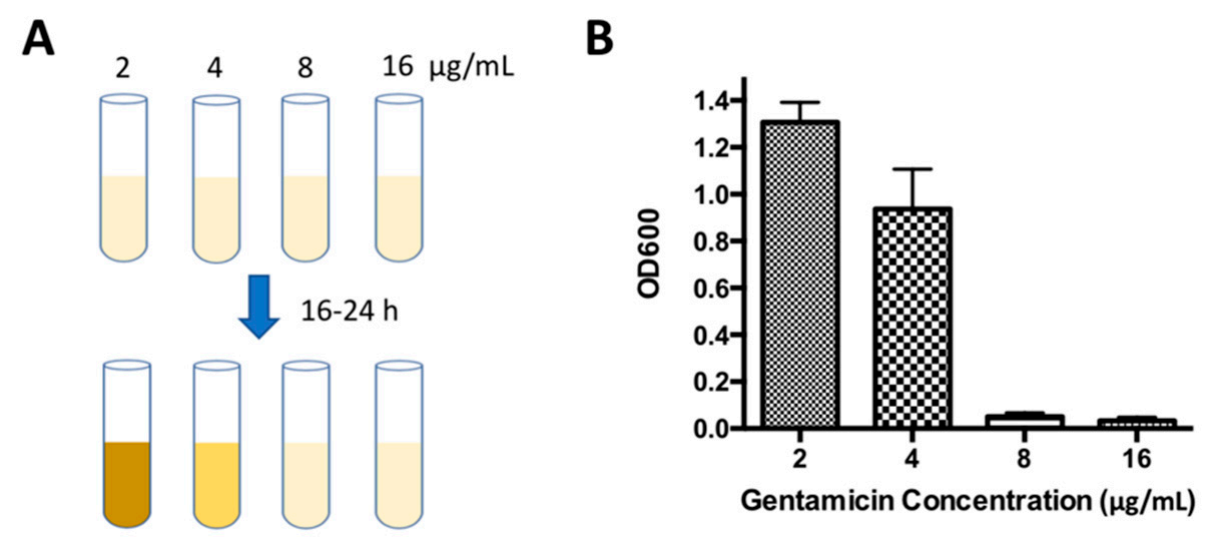
(**A**) Schematic illustration of MIC determination by broth dilution method. (**B**) OD600 obtained after the bacteria were incubated for 16 h.

To investigate the ability of our method to determine antibiotic susceptibility, we performed tests through tracking single-cell growth of *E. cloacae* in response to gentamicin treatments, which is illustrated in Scheme 1. Herein, the tested concentrations of gentamicin in gel pad were 2, 4, 8 and 16 μg/mL and the number of cells treated at each antibiotic concentration were 3964, 4387, 5199 and 5778, respectively. The populations were sufficiently large to show statistical significance in addressing the biological heterogeneity. Based on the analysis, results obtained from time-lapse images of the sample area captured at 0, 15, 30, 45 and 60 min, we found that the growth rates of the cells at each time point were highly diverse and displayed broad distributions. To simplify the analysis, we set integral bins for the growth rates and obtained cell count-based distributions shown in Figure 2. Assuming that the fraction of cells beyond the threshold of two were those that had been replicated, obviously, such fractions treated with 2 and 4 μg/mL gentamicin increased over time, suggesting that a majority of cells were continuously growing. In the groups treated with 8 and 16 μg/mL gentamicin, even though the replication of most of cells were arrested, a tiny portion of cells still survived and completed one replication. By specifically examining the growth rates of these cells, we learned that all of them were in a trend to stop growing by 60 min. Regarding the effectiveness of eventually inhibiting cell growth, as depicted in Figure 3, MIC could be determined as 8 μg/mL, which agreed with the result from conventional broth dilution method (Figure 4).

### 2.3. Rapid Antibiotic Susceptibility Determination of Phenotypically Heterogeneous Samples

**Figure 5:**
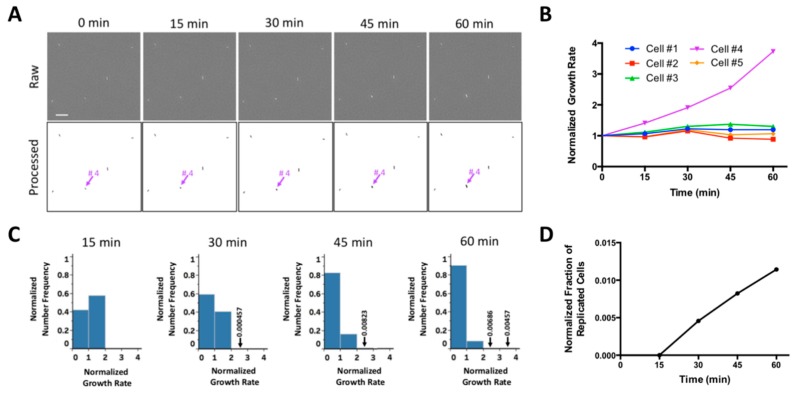
(**A**) Time-lapse images of one representative area, where kanamycin resistant *E. coli* and susceptible *E. cloacae* cells coexist in the presence of 50 µg/mL of kanamycin and corresponding post-processing binary images. Scale bar, 10 µm. Actively growing cells are marked with purple arrows. (**B**) Single-cell growth rate of bacterial cells shown in (**A**). (**C**) Number-based distribution of normalized single-cell growth rate for several thousand mixed bacterial cells treated with 50 µg/mL of kanamycin. (**D**) Normalized fraction of replicated cells versus time.

In practice, clinical bacteria isolates are complex because antibiotic resistant and susceptible bacteria coexist. Especially if the portion of the resistant phenotype is extremely small, accurately assessing the antibiotic susceptibility of such sample still remains challenging. Herein, our AST platform was further employed to analyze an artificial sample containing a mixture of kanamycin resistant *E. coli* and kanamycin susceptible *E. cloacae*, where the number of resistant cells was extremely low. Cells were treated with 50 μg/mL kanamycin, which was effective enough to selectively allow *E. coli* to proliferate, while killing *E. cloacae*. Figure 5A,B show a representative field of view selected from an area of 7.46 mm × 7.12 mm and the corresponding plot of normalized growth rate versus time. Apparently, only one out of five bacteria was actively growing. Depicted in Figure 5C are single-cell growth rate distributions for a total 2189 cells in the sample area at different time points. If a value of two is set as the threshold to separate replicated and non-replicated populations, as illustrated in Figure 5D, the fractions of replicated cells at 15, 30, 45 and 60 min are 0, 0.05%, 0.82% and 1.15%, respectively. In contrast, the majority remained as non-replicated and their size shrank over time, indicating that they were sensitive to kanamycin. Further examining each cell growth rate curve, confirmed that 25 cells (1.15% of total count) continuously multiplied while the others did not. Therefore, our method was able to distinguish antibiotic resistant phenotype from susceptible phenotype within 1 h, especially the portion of resistant subpopulation was as small as only about 1%. For comparison, conventional broth dilution was performed with sample volume of tested sample. The liquid medium turned turbid after 20 h incubation, suggesting that our approach was powerful in saving significant time without compromising the accuracy.

Despite the great performance in accurately determining the antibiotic susceptibility profiles for complicated bacterial samples, acquisition of a wide-field image containing several thousand cells takes about four minutes, testing multiple antibiotic concentrations simultaneously is still challenging. However, recently emerged ultrafast high-resolution WSI techniques [40,41,42] would raise the promise in solving this issue by completing the acquisitions of multiple wide-field images in one minute.

## 3. Materials and Methods

### 3.1. Materials and Instruments

Mueller-Hinton (MH) broth, Luria-Bertani (LB) broth, ampicillin sodium salt and gentamicin sulfate were purchased from Sigma-Aldrich (St. Louis, MO, USA). Agarose was obtained from Promega (Madison, WI, USA). Silicon wafer was purchased from University Wafer (Boston, MA, USA). Kanamycin sulfate, polystyrene petri dishes (diameter 100 mm) and glass microscope slides were purchased from Thermo Fisher Scientific (Waltham, MA, USA). *Enterobacter cloacae* (*E. cloacae*) (ATCC 13047) was purchased from the American Type Culture Collection (Manassas, VA, USA). A genetically engineered kanamycin resistant *Escherichia coli* (*E. coli*) strain was used as a model bacterium. Image acquisition was conducted using BZ-X800 All-in-One fluorescence microscope (Keyence Corporation, Osaka, Japan).

### 3.2. Bacterial Cell Culture

As the bacteria used in this study are resistant to certain antibiotics, corresponding antibiotics were added into the culture medium for the selection purpose. *E. cloacae* and kanamycin resistant *E. coli* were cultured in LB medium supplemented with 50 μg/mL ampicillin and 50 μg/mL kanamycin, respectively. All the antibiotics solutions were sterilized by being filtered through 0.2 μm polyvinylidene fluoride (PVDF) membrane (EMD Millipore, Burlington, MA, USA) prior to the addition to LB medium. After the stocked bacteria were inoculated in LB medium and pre-enriched in a 37 °C shaker for 3 h, the cells were diluted with phosphate buffered saline (PBS) to the desired concentrations for testing.

### 3.3. Sample Preparation

First, two parallel microscope glass slides with two pieces of spacers (0.38 mm-thick silicon wafer) in between were placed in a petri dish. Next, MH medium with 0.6% (*w/v*) agarose suspended in it was sterilized by autoclaving at 121 °C for 15 min. After the media cooled down below 50 °C, the antibiotic was added and this molten medium was subsequently poured into a petri dish and it filled the empty chamber between the glass slides. This petri dish was placed on a horizontal benchtop at room temperature. After the medium was sufficiently solidified, the cover glass slide was gently removed to present a thin gel pad with a flat surface. Then, 2.5 μL bacterial suspension was carefully loaded onto the gel pad without disrupting the surface. After the drop completely evaporated, the glass slide and the thin gel pad together was taken out by cutting off the surrounding bulk gel and was mounted at the side of the gel pad with the bacteria on an another glass slide for WSI.

### 3.4. Whole Slide Imaging

The aforementioned sandwiched glass slides were placed on the imaging stage of microscopy cell incubator, wherein the bacteria side of the gel was in contact with the supporting glass slide. After the horizontal plane of bacterial cells was focused by the objective lens, the boundary of sample area was determined by the auto-searching function of the Keyence microscope. Next, cells in 10 different field-of-views throughout the sample area were focused one by one to ensure the autofocusing during the scanning. Subsequently, tiles of phase contrast images were continuously captured as the sample holder stage moved along a sequential path programmed by the imager software until the entire sample area was scanned. Herein, tiles (0.362 mm × 0.272 mm for each tile) were acquired by a 40× objective lens (NA=0.60, S Plan Fluor ELWD Ph2, Nikon). Then, the BZ-X800 analyzer software automatically created a composite image by seamlessly stitching all the tiles together. After the first scanning, the temperature of the incubator was set to 37 °C. The scanning was repeated at 15, 30, 45 and 60 min after the temperature reached 37 °C.

### 3.5. Image Processing and Data Analysis

The images obtained from WSI were processed by ImageJ software version 1.52i (NIH, Bethesda, MD, USA). First, the threshold was adjusted to enhance the contrast of the bacterial cells and render cells as black and the background as white. Next, the area of each cell or microcolony developed from it was measured in pixels. Those below 15 pixels were filtered as they were considered as noise from the background. Then, these values of area were grouped according to the x-y coordinates on the image to deliver the change in size as each single cell grew over time. Finally, the normalized single-cell growth rate was calculated from the normalized change in size.

### 3.6. WSI-Based AST

To evaluate the accuracy of our WSI-based AST method in the determination of MIC, *E. cloacae* and gentamicin were used as a model bacterium and antibiotic, respectively. *E. cloacae* were treated with a series of concentrations (2, 4, 8 and 16 μg/mL) of gentamicin, generated in the gel pad of the bacterial culturing slides. The testing for each concentration was performed once using the aforementioned time-lapse WSI-based method to monitor the growth of each individual bacterial cell.

In addition, to assess the ability of our method in testing a polymicrobial and phenotypically heterogenous bacterial sample, we mixed kanamycin-sensitive *E. cloacae* and kanamycin-resistant *E. coli* together to stimulate the expected sample. Next, a WSI-based test was performed by treating the cells from this sample with 50 μg/mL kanamycin.

### 3.7. AST Using Broth Dilution Method

For comparison, AST was also performed by the gold standard broth dilution method [43]. In this work, the antibiotic was added to MH medium and was serially diluted to the desired concentrations. Subsequently, the same number of bacteria which was used for WSI-based test were inoculated into test tubes containing 5 mL MH medium with antibiotics and one tube of antibiotic-free medium as a control. After incubation at 37 °C for 16–24 h, the MIC was determined as the lowest concentration at which no visible bacterial growth was observed. Here, the bacterial growth was quantified by the measurement of OD600 using UV-Vis spectrophotometer (Nanodrop 1000, Thermo Fisher Scientific, Waltham, MA, USA). This test was conducted in triplicate.

## 4. Conclusions

In this study, we demonstrated a novel rapid and accurate AST method established on WSI, which enabled high-throughput analysis of single-cell growth rates regardless of the variations in the size and shape of bacteria. As a demonstration of employing this new method to perform AST, the MIC of *E. cloacae* against gentamicin was determined within the theoretically shortest time, which ensures that the growth of each individual bacterium is inhibited. Notably, our technique was able to rapidly identify antibiotic resistant cells from a large population of antibiotic susceptible cells, in which the portion of resistant subpopulation was about 1%. In addition, microscopy imaging shows no limitation in analyzing cells in diverse size or shape, suggesting that this method can be generalized to most bacteria species. Therefore, our AST approach would show great potential in determining antibiotic susceptibility of complex clinical bacteria isolates. Moreover, owing to the feature of high-throughput quantitative analysis, our approach can be applied to timely identifying the rising antibiotic resistance adapted to the treatment and guiding the adjustment of the treatment strategy to prevent the aggravation of antibiotic resistance.

## References

[1] Michael C.A., Dominey-Howes D., Labbate M. (2014). The antimicrobial resistance crisis: Causes, consequences, and management. Front Public Health.

[2] Boucher H.W., Talbot G.H., Bradley J.S., Edwards J.E., Gilbert D., Rice L.B., Scheld M., Spellberg B., Bartlett J. (2009). Bad bugs, no drugs: No ESKAPE! An update from the Infectious Diseases Society of America. Clin. Infect. Dis..

[3] Gajdacs M. (2019). The Continuing Threat of Methicillin-Resistant Staphylococcus aureus. Antibiotics.

[4] Ahmed M.O., Baptiste K.E. (2018). Vancomycin-Resistant Enterococci: A Review of Antimicrobial Resistance Mechanisms and Perspectives of Human and Animal Health. Microb. Drug Resist..

[5] Paterson D.L., Bonomo R.A. (2005). Extended-spectrum beta-lactamases: A clinical update. Clin. Microbiol. Rev..

[6] Jean S.S., Lee N.Y., Tang H.J., Lu M.C., Ko W.C., Hsueh P.R. (2018). Carbapenem-Resistant Enterobacteriaceae Infections: Taiwan Aspects. Front Microbiol..

[7] Gajdacs M. (2019). The Concept of an Ideal Antibiotic: Implications for Drug Design. Molecules.

[8] Silver L.L. (2011). Challenges of Antibacterial Discovery. Clin. Microbiol. Rev..

[9] Jorgensen J.H., Ferraro M.J. (2009). Antimicrobial susceptibility testing: A review of general principles and contemporary practices. Clin. Infect. Dis..

[10] Idelevich E.A., Silling G., Niederbracht Y., Penner H., Sauerland M.C., Tafelski S., Nachtigall I., Berdel W.E., Peters G., Becker K. (2015). Molecular Diagnostics of Sepsis Study, G., Impact of multiplex PCR on antimicrobial treatment in febrile neutropenia: A randomized controlled study. Med. Microbiol. Immunol..

[11] Kollef M.H. (2000). Inadequate antimicrobial treatment: An important determinant of outcome for hospitalized patients. Clin. Infect. Dis..

[12] Kumar A., Roberts D., Wood K.E., Light B., Parrillo J.E., Sharma S., Suppes R., Feinstein D., Zanotti S., Taiberg L. (2006). Duration of hypotension before initiation of effective antimicrobial therapy is the critical determinant of survival in human septic shock. Crit. Care Med..

[13] Kumar A., Ellis P., Arabi Y., Roberts D., Light B., Parrillo J.E., Dodek P., Wood G., Kumar A., Simon D.J.C. (2009). Initiation of inappropriate antimicrobial therapy results in a fivefold reduction of survival in human septic shock. Chest.

[14] Rolain J.M., Mallet M.N., Fournier P.E., Raoult D. (2004). Real-time PCR for universal antibiotic susceptibility testing. J. Antimicrob. Chemother..

[15] Sparbier K., Schubert S., Weller U., Boogen C., Kostrzewa M. (2012). Matrix-assisted laser desorption ionization-time of flight mass spectrometry-based functional assay for rapid detection of resistance against beta-lactam antibiotics. J. Clin. Microbiol..

[16] Vrioni G., Tsiamis C., Oikonomidis G., Theodoridou K., Kapsimali V., Tsakris A. (2018). MALDI-TOF mass spectrometry technology for detecting biomarkers of antimicrobial resistance: Current achievements and future perspectives. Ann. Transl. Med..

[17] Pulido M.R., Garcia-Quintanilla M., Martin-Pena R., Cisneros J.M., McConnell M.J. (2013). Progress on the development of rapid methods for antimicrobial susceptibility testing. J. Antimicrob. Chemother..

[18] Schoepp N.G., Khorosheva E.M., Schlappi T.S., Curtis M.S., Humphries R.M., Hindler J.A., Ismagilov R.F. (2016). Digital Quantification of DNA Replication and Chromosome Segregation Enables Determination of Antimicrobial Susceptibility after only 15 Minutes of Antibiotic Exposure. Angew. Chem. Int. Ed. Engl..

[19] Schoepp N.G., Schlappi T.S., Curtis M.S., Butkovich S.S., Miller S., Humphries R.M., Ismagilov R.F. (2017). Rapid pathogen-specific phenotypic antibiotic susceptibility testing using digital LAMP quantification in clinical samples. Sci. Transl. Med..

[20] Gajdacs M., Spengler G., Urban E. (2017). Identification and Antimicrobial Susceptibility Testing of Anaerobic Bacteria: Rubik’s Cube of Clinical Microbiology?. Antibiotics.

[21] Hughes D., Andersson D.I. (2017). Environmental and genetic modulation of the phenotypic expression of antibiotic resistance. FEMS Microbiol. Rev..

[22] Grobner S., Dion M., Plante M., Kempf V.A. (2009). Evaluation of the BD GeneOhm StaphSR assay for detection of methicillin-resistant and methicillin-susceptible Staphylococcus aureus isolates from spiked positive blood culture bottles. J. Clin. Microbiol..

[23] Syal K., Mo M., Yu H., Iriya R., Jing W., Guodong S., Wang S., Grys T.E., Haydel S.E., Tao N. (2017). Current and emerging techniques for antibiotic susceptibility tests. Theranostics.

[24] Leonard H., Colodner R., Halachmi S., Segal E. (2018). Recent Advances in the Race to Design a Rapid Diagnostic Test for Antimicrobial Resistance. ACS Sens..

[25] Choi J., Jung Y.G., Kim J., Kim S., Jung Y., Na H., Kwon S. (2013). Rapid antibiotic susceptibility testing by tracking single cell growth in a microfluidic agarose channel system. Lab. Chip..

[26] Choi J., Yoo J., Lee M., Kim E.G., Lee J.S., Lee S., Joo S., Song S.H., Kim E.C., Lee J.C. (2014). A rapid antimicrobial susceptibility test based on single-cell morphological analysis. Sci. Transl. Med..

[27] Choi J., Jeong H.Y., Lee G.Y., Han S., Han S., Jin B., Lim T., Kim S., Kim D.Y., Kim H.C. (2017). Direct, rapid antimicrobial susceptibility test from positive blood cultures based on microscopic imaging analysis. Sci. Rep..

[28] Baltekin O., Boucharin A., Tano E., Andersson D.I., Elf J. (2017). Antibiotic susceptibility testing in less than 30 min using direct single-cell imaging. Proc. Natl. Acad. Sci. USA.

[29] Cermak N., Olcum S., Delgado F.F., Wasserman S.C., Payer K.R., Murakami M.A., Knudsen S.M., Kimmerling R.J., Stevens M.M., Kikuchi Y. (2016). High-throughput measurement of single-cell growth rates using serial microfluidic mass sensor arrays. Nat. Biotechnol..

[30] Kang W., Sarkar S., Lin Z.S., McKenney S., Konry T. (2019). Ultrafast Parallelized Microfluidic Platform for Antimicrobial Susceptibility Testing of Gram Positive and Negative Bacteria. Anal. Chem..

[31] Davies N.G., Flasche S., Jit M., Atkins K.E. (2019). Within-host dynamics shape antibiotic resistance in commensal bacteria. Nat. Ecol. Evol..

[32] Colijn C., Cohen T., Fraser C., Hanage W., Goldstein E., Givon-Lavi N., Dagan R., Lipsitch M. (2010). What is the mechanism for persistent coexistence of drug-susceptible and drug-resistant strains of Streptococcus pneumoniae?. J. Soc. Interface..

[33] Salipante S.J., Sengupta D.J., Rosenthal C., Costa G., Spangler J., Sims E.H., Jacobs M.A., Miller S.I., Hoogestraat D.R., Cookson B.T. (2013). Rapid 16S rRNA next-generation sequencing of polymicrobial clinical samples for diagnosis of complex bacterial infections. PLoS ONE.

[34] Cummings L.A., Kurosawa K., Hoogestraat D.R., SenGupta D.J., Candra F., Doyle M., Thielges S., Land T.A., Rosenthal C.A., Hoffman N.G. (2016). Clinical Next Generation Sequencing Outperforms Standard Microbiological Culture for Characterizing Polymicrobial Samples. Clin. Chem..

[35] Young K.D. (2006). The selective value of bacterial shape. Microbiol. Mol. Biol. Rev..

[36] Al-Janabi S., Huisman A., Van Diest P.J. (2012). Digital pathology: Current status and future perspectives. Histopathology.

[37] Abels E., Pantanowitz L. (2017). Current State of the Regulatory Trajectory for Whole Slide Imaging Devices in the USA. J. Pathol. Inform..

[38] Ghaznavi F., Evans A., Madabhushi A., Feldman M. (2013). Digital imaging in pathology: Whole-slide imaging and beyond. Ann. Rev. Pathol..

[39] Song D., Liu H., Dong Q., Bian Z., Wu H., Lei Y. (2018). Digital, Rapid, Accurate, and Label-Free Enumeration of Viable Microorganisms Enabled by Custom-Built On-Glass-Slide Culturing Device and Microscopic Scanning. Sensors.

[40] Liao J., Wang Z., Zhang Z., Bian Z., Guo K., Nambiar A., Jiang Y., Jiang S., Zhong J., Choma M. (2018). Dual light-emitting diode-based multichannel microscopy for whole-slide multiplane, multispectral and phase imaging. J. Biophotonics..

[41] Liao J., Jiang Y., Bian Z., Mahrou B., Nambiar A., Magsam A.W., Guo K., Wang S., Cho Y.K., Zheng G. (2017). Rapid focus map surveying for whole slide imaging with continuous sample motion. Opt. Lett..

[42] Liao J., Jiang S., Zhang Z., Guo K., Bian Z., Jiang Y., Zhong J., Zheng G. (2018). Terapixel hyperspectral whole-slide imaging via slit-array detection and projection. J. Biomed. Opt..

[43] Wiegand I., Hilpert K., Hancock R.E. (2008). Agar and broth dilution methods to determine the minimal inhibitory concentration (MIC) of antimicrobial substances. Nat. Protoc..

